# Complex effects of temperature on mosquito immune function

**DOI:** 10.1098/rspb.2012.0638

**Published:** 2012-05-16

**Authors:** C. C. Murdock, Krijn P. Paaijmans, Andrew S. Bell, Jonas G. King, Julián F. Hillyer, Andrew F. Read, Matthew B. Thomas

**Affiliations:** 1 Department of Entomology, Center for Infectious Disease Dynamics, Merkle Lab, University Park, PA 16802, USA; 2 Department of Biology, Center for Infectious Disease Dynamics, Millennium Science Complex, University Park, PA 16802, USA; 3 Department of Biological Sciences and Institute for Global Health, Vanderbilt University, VU Station B 35-1634, Nashville, TN 37235, USA

**Keywords:** mosquito, innate immunity, temperature, vector, parasite

## Abstract

Over the last 20 years, ecological immunology has provided much insight into how environmental factors shape host immunity and host–parasite interactions. Currently, the application of this thinking to the study of mosquito immunology has been limited. Mechanistic investigations are nearly always conducted under one set of conditions, yet vectors and parasites associate in a variable world. We highlight how environmental temperature shapes cellular and humoral immune responses (melanization, phagocytosis and transcription of immune genes) in the malaria vector, *Anopheles stephensi. Nitric oxide synthase* expression peaked at 30°C, *cecropin* expression showed no main effect of temperature and humoral melanization, and phagocytosis and *defensin* expression peaked around 18°C. Further, immune responses did not simply scale with temperature, but showed complex interactions between temperature, time and nature of immune challenge. Thus, immune patterns observed under one set of conditions provide little basis for predicting patterns under even marginally different conditions. These quantitative and qualitative effects of temperature have largely been overlooked in vector biology but have significant implications for extrapolating natural/transgenic resistance mechanisms from laboratory to field and for the efficacy of various vector control tools.

## 1. Introduction

During the last decade, considerable effort has been devoted to elucidating the molecular and cellular interactions between mosquitoes and a range of parasites and pathogens [1–5]. This research has advanced general knowledge of innate immune systems [6], and identified key mosquito immune genes, effector molecules and defence pathways that can decrease or block the development of key vector-borne disease agents, providing potential targets for transgenic manipulation [7–11].

Even though the current reductionist paradigm of vector immunology has been extremely insightful, this approach is incomplete. Mosquito resistance to infection is not a static phenotype comprised solely of immune genes involved in standard immune responses measured under customary laboratory conditions [12,13]. Hosts and parasites associate in a variable world. Vector competence involves broad aspects of host physiology and condition, which is shaped by both genetic and environmental variation that often interact in nonlinear ways [14]. From work in other invertebrate–parasite systems, small, realistic changes in temperature can have striking effects on the outcome of invertebrate host–parasite interactions. Ambient temperature profoundly affects overall resistance to a wide diversity of parasites: viruses [15,16], bacteria [17,18], microsporidia [19], fungi [20–22], nematodes [23] and parasitoids [24,25]. Temperature also influences the duration of latency periods [26] and time to host recovery [19].

This evidence suggests that mosquitoes should exhibit diverse resistance phenotypes across different ambient temperatures. Temperature may shape the resistance phenotype and parasite growth in two ways: (i) direct effects of host body temperature on parasite growth (which are independent of the mosquito host), and (ii) the less well studied indirect effects on parasite growth, which are mediated through temperature effects on mosquito innate immune mechanisms. Yet the majority of studies examining aspects of mosquito immune function are conducted under standard laboratory conditions using single temperatures and often single time points for assessing experimental read-outs. Paradoxically, we know more about how environmental variability shapes the immune phenotype in butterflies [27,28], fruitflies [18,29], crickets [30], meal worms [31] and moths [32] than we do in most disease vectors. Given the global health and economic burdens imposed by vector-borne parasites such as malaria, this represents a significant knowledge gap.

If the relative and/or absolute immune response of mosquitoes exhibits thermal sensitivity, the current approach of outlining innate immune responses under standard laboratory conditions is insufficient for understanding vector competence as played out in the field. To test this assertion, we measured humoral and cellular immune responses across a range of different, constant temperatures in the Asian malaria vector, *Anopheles stephensi*. We demonstrate that temperature can have dramatic and diverse quantitative and qualitative impacts on mosquito immune responses, with potentially complex interactions with factors such as time and nature of immune challenge. That immune responses are affected by temperature is not necessarily surprising. That the effects are complex and unpredictable across different immune measures represents a challenge to current disciplinary convention, where environmental variation is generally ignored.

## 2. Methods

### (a) Mosquito rearing and handling

We reared *An. stephensi* (Liston) under standard insectary conditions at 27 ± 1°C, 80 per cent humidity and a 12 L : 12 D photo-period. We placed mosquito eggs into plastic trays (25 × 25× 7 cm) filled with 1.5 l of water. To minimize any potential variation in emerging adult mosquito body size, we divided recently hatched larvae to ensure a density of 400 individuals per tray. Larvae were fed Liquifry for the first 5 days post-hatching, and then were fed Tetrafin fish flakes for the duration of the larval period. Pupae were collected from larval trays and placed into experimental cages approximately two weeks after egg hatch. Upon emergence, adults were fed ad libitum on a 6 per cent glucose solution. Mosquitoes used for humoral melanization and immune gene expression experiments were provided a bloodmeal from rats (Wistar, more than six weeks old) at 3 days post-emergence. On day 3–4 post-emergence, mosquitoes were anaesthetized on ice and the immune challenge administered by an intrathoracic injection into the anepisternal cleft [33] with a mouth pipette and microcapillary glass needle or a Nanoject. After immune challenge, mosquitoes were randomly assigned to one of five reach-in incubators with temperatures of 12°C, 18°C, 24°C, 28°C and 34 ± 0.5°C; relative humidity 80 ± 5%. A series of pilot experiments for each immune measure was conducted across a reduced temperature and a sampling time point regime to confirm that the effects of temperature, immune challenge and sampling time point on immune responsiveness were consistent in the full experiment (see electronic supplementary material, text S1).

### (b) Melanization: immune challenge with Sephadex beads

Melanization is the product of a series of enzymatic and non-enzymatic reactions beginning with the hydroxylation of tyrosine and ending with the oxidate polymerization of indolequinones [34]. To date, many studies have used total phenoloxidase activity, a key enzyme in the melanization reaction, as a proxy for immunocompetence [27,35–37]. However, because phenoloxidases are involved in a variety of other metabolic functions in addition to innate immunity [34], we chose to measure the melanization response directly. Melanization has been implicated in the defences of refractory *Anopheles gambiae* (L35) strain against oocysts of the rodent malaria *Plasmodium berghei* [38–40] and new world *Plasmodium falciparum* [41]*, Aedes aegypti* against *Plasmodium gallinaceum* sporozoites [42], *Ae. aegypti* and *Armigeres subalbautus* against bacteria [42,43], and *Ar. subalbatus* against filarial worms [44,45]. To stimulate the melanization response, we injected blood-fed females with one negatively charged CM-25 Sephadex bead. Sephadex beads range in size from 40 to 120 μm in diameter, and only the smallest beads were selected visually for inoculation. Beads were suspended in a DMEM solution (Dulbecco's Modification of Eagle's Modification) and 0.001 per cent methyl green to facilitate bead visualization [46]. We injected one bead in a minimal amount of solution (less than 0.5 μl) and randomly distributed mosquitoes across temperature treatments. At 24 hours post-immune challenge mosquitoes that were able to walk were removed, and beads were dissected out in a phosphate-buffered saline solution stained with 0.01 per cent methyl green.

### (c) Phagocytosis: immune challenge with fluospheres

Phagocytosis is a cellular immune response that involves haemocyte recognition, engulfing and destruction of small micro-organisms and apoptotic cells. Phagocytosis is an evolutionarily conserved immune response that plays important roles in antibacterial defence [47]. To stimulate phagocytosis, we injected non-blood-fed females with approximately 50 000 yellow-green carboxylate-modified fluospheres (1 μm diameter) with a Nanoject. After immune challenge, 10 mosquitoes were randomly allocated to a temperature treatment and one of four sampling time points (1, 6, 12 and 24 h). At 1–24 h post-immune challenge, mosquitoes were removed and haemocytes were fluorescently stained *in vivo* by injecting each mosquito with a solution of Hoescht nucleic acid stain and Vybrant CM-DiI cell-labelling solution (Invitrogen Life Technologies, Carlsbad, CA). Haemolymph was then collected by perfusion from ice-anaesthetized mosquitoes [48] onto a microscope slide. Haemocytes were fixed in 4 per cent paraformaldehyde, washed in phosphate-buffered saline solution (pH 7.4, 0.2 M) and distilled water, and mounted with Aqua-Poly/Mount. For each mosquito, we calculated the phagocytic index and the phagocytic capacity for a total of 50 counted granulocytes [49].

### (d) Gene expression: immune challenge with bacteria

We investigated the effects of temperature on *defensin 1* (*DEF1*), *cecropin 1* (*CEC1*) and *nitric oxide synthase* (*NOS*) gene expression in response to no manipulation, injury or heat-killed *Escherichia coli* challenge. *DEF1* and *CEC1* encode two antimicrobial peptides that are produced in the insect fat body and by local barrier epithelia. *DEF1* is active against Gram-positive bacteria and filamentous fungi [50], *CEC1* is active against both Gram-positive and -negative bacteria [51], and both peptides have been implicated to some extent with *Plasmodium* killing [10,52]. *NOS* encodes nitric oxide, an effector molecule that has been shown to be a ubiquitous killer of a wide diversity of pathogens and parasites [53], and has also been implicated as a major anti-malarial defence in the mosquito midgut epithelia [54–57].

We used heat-killed tetracycline-resistant GFP-expressing *E. coli* (dh5 alpha strain) as our challenge to avoid temperature-mediated variation in bacterial growth within mosquitoes housed at different mean temperatures. *Escherichia coli* were grown overnight in Luria-Bertani's rich nutrient medium (LB) in a shaking incubator at 37°C, and a serial dilution was prepared from the overnight culture. To approximate our injection dose of *E. coli*, we recorded the absorbance (OD_600_) from each dilution with a NanoDrop (Thermo Scientific, Wilmington, DE). To estimate the dose of *E. coli*, we compared the absorbance of each dilution to a standard curve of the linear relationship between absorbance and colony-forming units (CFUs) of *E. coli* that was generated prior to the experiment. The dilution with an absorbance corresponding to approximately 1 × 10^9^ *E. coli* per millilitre (i.e. 200 000 bacteria per injection) was selected for our injection stock. To further confirm this estimate, we plated our injection stock in triplicate onto LB agar plates, placed them overnight into an incubator at 37°C, and counted the resulting CFUs the next day. We then killed the *E. coli* stock by autoclaving for 25 min. Ice-anaesthetized mosquitoes were either unmanipulated (control mosquitoes), or received an injection of either 0.2 μl of sterile LB (positive injury control) or 200 000 heat-killed *E. coli* before being placed into their respective temperature treatment. Fifteen mosquitoes from each immune-challenge group were then allocated to each of five temperatures and four sampling sessions (6, 12, 18 and 24 h).

### (e) RNA collection, cDNA synthesis and quantitative PCR

Post-immune challenge, mosquitoes were removed from their temperature treatment, killed with chloroform and immediately stored in RNA*later* RNA stabilization reagent at 4°C for future molecular analyses. Immediately after the termination of the experiment, five mosquitoes from each treatment group (*n* = 300 total) were isolated individually in β-Mercaptoethanol and RLT lysis buffer. Messenger RNA was extracted using the Qiagen RNeasy Mini Kit for animal tissues (as per the manufacturer's protocol). Standards for quantitative polymerase chain reaction (PCR) were prepared by extracting mRNA from a pool of four mosquitoes. The concentration of mRNA in each sample was quantified with a NanoDrop and stored at −80°C. RNA was converted to cDNA with a high-capacity cDNA reverse transcription kit as per the manufacturer's protocol (Applied Biosystems, Foster City, CA) on a Mastercycler Gradient thermal cycler (Eppendorf, Hamburg, Germany).

The expression of *ribosomal protein S7*, a standard housekeeping gene in mosquito gene expression studies [57–61], was influenced by experimental treatment (see electronic supplementary material, text S2, table S2.2 and figure S2.2). Owing to concerns that the expression of other housekeeping genes may also be influenced by temperature (as reflected by the effects of temperature on total RNA concentration; see electronic supplementary material, text S2, table S2.2 and figure S2.2), we chose to quantify our diluted cDNA from our experimental samples by comparing their threshold cycle numbers against a standard curve generated from 1 : 10 serial dilutions of our standard sample (cDNA from a pool of four mosquitoes; see electronic supplementary material, text S3). Three replicates of each cDNA standard spanning six orders of magnitude were included in each quantitative PCR run. We measured cDNA counts for each gene of interest from individual mosquitoes relative to the standard curve of that assay. DNA contamination in RNA samples was confirmed to be undetectable using quantitative PCR, and primers and probes were designed from *An. stephensi* and *An. gambiae* sequences (see electronic supplementary material, text S3).

### (f) Statistical analyses

All statistical analyses for these experiments were run in PSAW 18.0 (IBM Corporation, New York, NY). Full models from generalized linear model (GLM) analysis were reduced through backward elimination of non-significant interactions. We assessed goodness of fit of the final models through model deviance, log likelihood values and Akaike information criterion. Covariates included in GLMs were centred on their grand mean.

#### (i) Humoral melanization: degree of bead melanization

We scored recovered beads for the degree of melanization by assigning each bead to one of three categorical classes: unmelanized, partially melanized (i.e. portions of the bead remained unmelanized) and fully melanized [62–64]. We ran a logistic regression to estimate how the probability of a bead being in a particular class was affected by temperature with total bead area as a covariate.

#### (ii) Phagocytosis: phagocytic index and capacity

We used GLMs to assess how temperature and sampling time point affected the proportion of phagocytizing granulocytes and the mean number of beads granulocytes can uptake. For both response variables, models included temperature, sampling time point and their interaction as fixed factors. The centred phagocytic index was included in the phagocytic capacity GLM as a covariate to account for a potential relationship between the number of active granulocytes (with beads) and the average number of beads granulocytes consume. We predicted estimated marginal means of phagocytic index and capacity assuming a normal distribution with identity link function and a Poisson distribution with log link function, respectively.

#### (iii) Gene expression

To compare differences in average gene expression among our treatment groups, we used the cDNA counts generated for each target gene from our standard curve analysis as our expression measure. We analysed all expression data with GLMs assuming a gamma distribution for the dependent variable, which was transformed with a log link function. Full factorial analyses were run for each gene separately to control for any differences in efficiencies among our assays as well as independence among our experimental samples. Temperature, sampling time point and immune challenge were included in all models as fixed factors. We included *rpS7* cDNA counts and the total RNA concentration of each sample as covariates in all models to adjust our estimated means of our target gene by any differences in baseline expression among mosquitoes. Inclusion of these covariates improved model fit, but the overall patterns of target gene expression were qualitatively similar without the covariates.

## 3. Results

We investigated whether the rates of characteristic humoral and cellular immune responses of insects were temperature-sensitive, and especially whether immune responses were influenced in qualitatively consistent ways across different immune challenges and sampling time points. Because temperature has been shown to influence pathogen performance [65–68], we use non-living immune stimuli in the subsequent experiments to disentangle the effects of temperature on immune performance.

### (a) Humoral melanization

**Figure 1.**
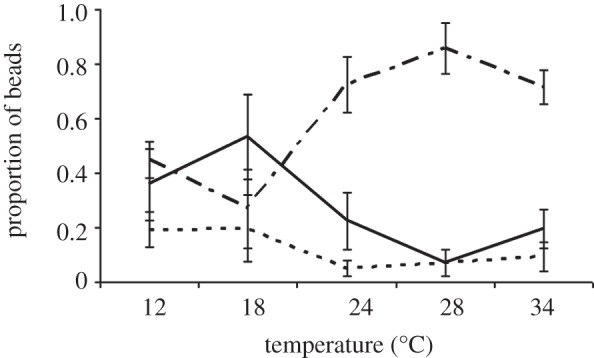
Temperature significantly influences the humoral melanization of Sephadex beads (logistic regression analysis: *n* = 136; χ*^2^* = 17.468, *p* = 0.004). Data show mean (±s.e.m.) proportion of unmelanized (dashed line), partially (broken line) and fully melanized beads (solid line) recovered at different temperatures 24 h post-injection. Even though more partially melanized beads were recovered at warmer temperatures, the probability of recovering fully melanized beads was highest at 18°C, suggesting that the rate of melanization is higher at cooler temperatures.

We recovered 98 per cent of injected beads from mosquitoes housed at all experimental temperatures. Temperature significantly affected the probability of recovering unmelanized, partially melanized or fully melanized beads (figure 1; *n* = 136; χ*^2^* = 17.468, *p* = 0.004). We recovered more fully melanized beads than partially melanized beads at 18°C (odds ratio 5.1) than at any other temperature. The proportion of partially melanized beads relative to fully melanized beads increased with temperature and peaked at 28°C (odds ratio 10.1). In contrast, neither temperature nor bead size (area) affected the probability of recovering unmelanized beads, and the size of the injected bead (bead area) did not significantly predict bead status. Peak rate of melanization appears to occur at 18°C and becomes less efficient at warmer temperatures (figure 1).

### (b) Phagocytosis

**Figure 2.**
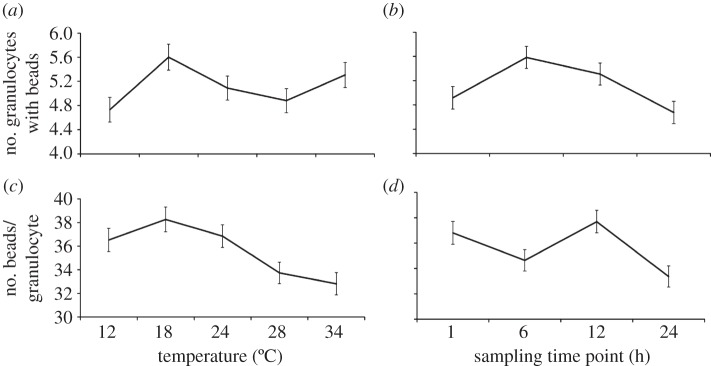
Both temperature and sampling time point significantly influenced the phagocytosis of fluospheres. The effects of temperature on the mean (±s.e.m.) phagocytic index (number of haemocytes containing fluospheres out of 50 counted haemocytes) and capacity (the number of beads per haemocyte) are represented by (*a*) and (*c*), respectively (GLMs: index, Poisson distribution with log link function, Wald 
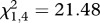
, *n* = 191, *p* < 0.0001; capacity, normal distribution with identity link function, Wald 
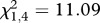
, *n* = 195, *p* = 0.026). (*b*) and (*d*) depict the effects of sampling time point on the mean (±s.e.) phagocytic index and capacity, respectively (GLMs: index, Poisson distribution with log link function, Wald 
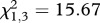
, *n* = 191, *p* = 0.001; capacity, normal distribution with identity link function, Wald 
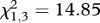
, *n* = 195, *p* = 0.002).

A GLM indicated that temperature significantly affected both phagocytic index (Poisson distribution with log link function: Wald 
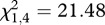
, *n* = 191, *p* < 0.0001; figure 2*a*) and capacity (normal distribution with identity link function: Wald 
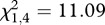
, *n* = 195, *p* = 0.026; figure 2*b*). The mean number of granulocytes phagocytizing fluorescent beads was significantly higher in mosquitoes housed at 18°C relative to mosquitoes housed at 28°C (Bonferroni-adjusted post hoc test: *p* = 0.010) and 34°C (Bonferroni-adjusted post hoc test: *p* = 0.001); mosquitoes housed at 34°C had a significantly lower phagocytic index than mosquitoes housed at cooler temperatures (Bonferroni-adjusted post hoc tests: 12°C versus 34°C, *p* = 0.046; 18°C versus 34°C, *p* = 0.001; 24°C versus 34°C, *p* = 0.022; figure 2*a*). Haemocytes consumed on average more beads in mosquitoes housed at 18°C than 12°C (Bonferroni-adjusted post hoc test: *p* = 0.032). However, there were no significant differences in the phagocytic capacity of haemocytes in mosquitoes housed at other temperatures.

Both the phagocytic index (GLM, Poisson distribution with log link function: Wald 
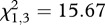
, *n* = 191, *p* = 0.001) and capacity (GLM, normal distribution with identity link function: Wald 
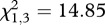
, *n* = 195, *p* = 0.002) were significantly influenced by sampling time point (figure 2). The mean number of granulocytes with beads varied across sampling time points (figure 2*c*), while the mean number of beads granulocytes consumed was highest 6–12 h post-immune challenge (Bonferroni-adjusted post hoc test: *p* = 0.002; figure 2*d*). There was a strong positive relationship between the phagocytic index and mean phagocytic capacity (Wald 
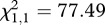
, *n* = 195, *p* < 0.0001; regression analysis controlling for the effects of temperature and sampling time: 
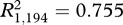
, *F* = 593.79, *p* < 0.0001). This suggests that immune stimulation of phagocytosis increased granulocyte efficiency, as well as overall activity, within the haemolymph. There was no significant interaction between temperature and sampling time point for either measure of phagocytosis.

### (c) *Defensin* expression

**Figure 3.**
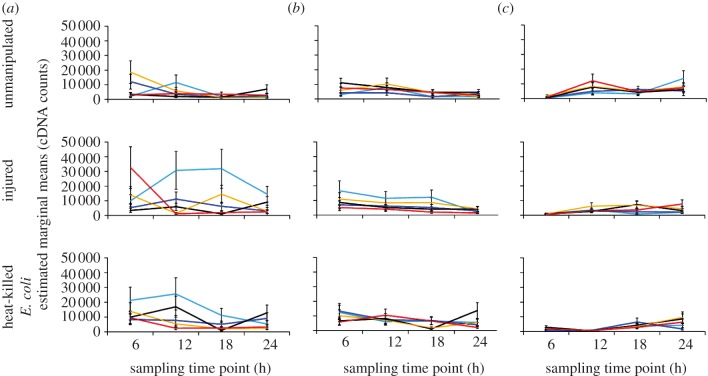
The effects of temperature, sampling time point and immune challenge on the expression of immune genes. The relationship between mean gene expression (cDNA counts ± s.e.m.) for (*a*) *defensin* (*DEF1*) and (*c*) *nitric oxide synthase* (*NOS*) and temperature varied significantly among mosquitoes sampled at different time points post-challenge and treated with different immune stimuli (GLM, gamma distribution with log link function: *DEF1* Wald 
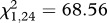
, *n* = 299, *p* < 0.0001; *NOS* Wald 
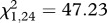
, *n* = 299, *p* = 0.003). The relationship between (*b*) *cecropin* (*CEC1*) expression and temperature varied significantly only among mosquitoes receiving different immune challenges (GLM, gamma distribution with log link function: Wald 
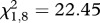
, *n* = 299, *p* = 0.004). Light blue lines, 18°C; dark blue, 22°C; black, 26°C; yellow, 30°C; red, 34°C.

Temperature significantly influenced the expression of *DEF1*; these effects were strongly shaped by sampling time point and immune challenge (table 1). For example, mosquitoes housed at 26°C experienced increased *DEF1* expression within the first 6–12 h and at 24 h post-immune challenge with either an injury or injection of heat-killed *E. coli*. However, this pattern is not maintained in mosquitoes housed at different temperatures that received the same immune challenge. In injured mosquitoes housed at 18°C, *DEF1* expression peaks 12–18 h post-immune challenge, while *DEF1* expression peaks within the first 6 h and rapidly declines at subsequent sampling time points in mosquitoes housed at 34°C (figure 3*a*). Alternatively, for mosquitoes treated with heat-killed *E. coli*, *DEF1* expression is elevated within the first 6 h for mosquitoes housed at warmer temperatures (30°C and 34°C), while *DEF1* expression is elevated in the first 6–12 h and declines thereafter in mosquitoes housed at 18°C. In addition to the interacting effects of temperature, sampling time point and immune challenge, there was a significant main effect of temperature on *DEF1* expression (table 1); mosquitoes housed at 18°C expressed considerably more *DEF1*, overall, relative to mosquitoes housed at warmer temperatures (Bonferroni-adjusted post hoc tests: 18°C versus 22°C, *p* = 0.037; 18°C versus 26°C, *p* = 0.001; 18°C versus 30°C, *p* = 0.002; and 18°C versus 34°C, *p* < 0.0001).

**Table 1.** Final model results for *DEF1*, *CEC1* and *NOS* from GLM analysis. A gamma distribution and log link function were assumed for all models. Dashes indicate higher order interactions backward eliminated from the full model. Omnibus tests confirmed that each fitted model was significantly different from its null model (*DEF1*: likelihood ratio 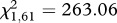, *p* < 0.0001; *CEC1*: likelihood ratio 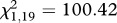, *p* < 0.0001; *NOS1*: likelihood ratio 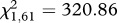, *p* < 0.0001). Goodness of fit was assessed by evaluating potential overdispersion through model deviance scores (*DEF1*: deviance value/d.f. = 1.25; *CEC1*: deviance value/d.f. = 1.11; *NOS*: deviance value/d.f. = 1.00). *p*-values are significant (in bold) if they were below a 0.05 probability of committing a Type I error.)

	*DEF1*			*CEC1*			*NOS*		
factors (*n* = 299)	d.f.	Wald χ^2^	*p*-value	d.f.	Wald χ^2^	*p*-value	d.f.	Wald χ^2^	*p*-value
intercept	**1**	**24954.69**	**<0.0001**	**1**	**24468.06**	**<0.0001**	**1**	**25699.21**	**<0.0001**
temperature	**4**	**34.84**	**<0.0001**	4	8.17	0.085	**4**	**20.04**	**<0.0001**
sampling time point	**3**	**42.31**	**<0.0001**	**3**	**31.68**	**<0.0001**	**3**	**142.27**	**<0.0001**
immune challenge	**2**	32.12	**<0.0001**	**2**	**11.12**	**0.004**	**2**	**18.14**	**<0.0001**
centred rpS7 cDNA counts	**1**	**4.76**	**0.029**	**1**	**6.76**	**0.009**	**1**	**4.88**	**0.027**
total RNA concentration	**1**	**4.91**	**0.027**	**1**	**5.41**	**0.020**	**1**	**145.54**	**<0.0001**
temperature × sampling time point	**12**	**76.66**	**<0.0001**	—	—	—	12	17.47	0.133
sampling time point × immune challenge	6	10.88	0.092	—	—	—	6	4.87	0.772
temperature × immune challenge	**8**	**26.59**	**0.001**	**8**	**22.45**	**0.004**	**8**	**111.01**	**<0.0001**
temperature × sampling time point × immune challenge	**24**	**68.56**	**<0.0001**	—	—	—	**24**	**47.23**	**0.003**

### (d) *Cecropin* expression

Temperature also significantly influenced the expression of *CEC1*, and this occurred in a manner which depended on the nature of the immune challenge (table 1). Unlike *DEF1* expression, the effects of temperature did not depend on the time of sampling. Generally, *CEC1* expression was highest in unmanipulated mosquitoes housed at optimal to warmer temperatures (26°C, 30°C and 34°C), injured mosquitoes housed at 18°C and 30°C, and heat-killed *E. coli*-treated mosquitoes housed at cooler to optimal temperatures (18°C, 22°C and 26°C; figure 3*b*).

### (e) *Nitric oxide synthase* expression

Similar to *DEF1* expression, the effects of temperature on *NOS* expression varied significantly with both sampling time point and immune challenge (table 1). In unmanipulated mosquitoes, *NOS* expression peaked at later sampling time points in mosquitoes housed at cooler temperatures (18°C: 24 h; 22°C: 18 h) than in mosquitoes housed at optimal or warmer temperatures (26–34°C: 12 h; figure 3*c*). Mosquitoes challenged with heat-killed *E. coli* generally experienced increased *NOS* expression 24 h post-immune challenge (with the exception of mosquitoes housed at 22°C; figure 3*c*). The relationship between sampling time and *NOS* expression is much more variable in injured mosquitoes housed at different temperatures (figure 3*c*). There also was a main effect of temperature on *NOS* expression (table 1); mosquitoes housed at 30°C had on average higher *NOS* expression than mosquitoes from other temperatures (Bonferroni-adjusted post hoc tests: 18°C versus 30°C, *p* = 0.002; 22°C versus 30°C, *p* = 0.016).

## 4. Discussion

Research on a range of insects and other ectotherms clearly demonstrates impacts of temperature on host resistance and parasite virulence. Here, we extend this research to show that ambient temperature can profoundly influence the rates of both humoral and cellular immune responses in a major malaria vector. Surprisingly, the effects of temperature do not simply scale quantitatively, nor are they consistent across immune measures. Accordingly, the standard approach of exploring immune function and mosquito–pathogen interactions under a very narrow range of temperatures in the laboratory fails to describe much of the immune phenotype relevant to more diverse field conditions. Further, for several of the measures, there were significant time or rate effects, which varied depending on the nature of immune challenge and/or complex interactions among factors (figure 3). The standard approaches that constrain such experimental complexity will miss these relevant intricacies.

A null hypothesis is that temperature effects on immune function should scale simply with temperature-related changes in general physiology and baseline gene expression. This is what we found for *CEC* expression, where there was no main effect of temperature above the background effects on housekeeping gene expression. However, *CEC* expression did vary with temperature depending on whether an injury or heat-killed *E. coli* were administered. Thus, while temperature initially appeared to be insignificant, interactions with other sources of ‘environmental’ variability can yield unpredictable and complex responses. Recent results from another insect system reinforce this finding, with temperature effects on innate immune measures manifesting only through complex interactions with other environmental variables, like density of conspecifics and quality of food resources [32].

*NOS* expression peaked slightly above the assumed temperature optimum for the mosquito; colonies are typically maintained at around 27°C, which is the optimum for other anophelines [69]. Nitric oxide functions as a cell signalling and cytotoxic effector molecule, and has been implicated as a major anti-malarial defence in the midgut of *An. stephensi*, contributing to the parasite bottleneck associated with ookinete migration through the midgut epithelium [54,70]. Further, it may also be a late-stage line of defence against *Plasmodium* parasites [55,56], with elevated activity being detected in the fat body as well as circulating granulocytes in response to infection [33]. Recent theoretical temperature models predict that the temperature optima for development of *P. falciparum* [71,72] and *P. vivax* [71] is around 30–31°C. Thus, increased expression of *NOS* at warmer temperatures may be an important mosquito defence that counters and limits optimal parasite development.

Unexpectedly, several of the immune responses studied were more robust at 18°C. However, evidence from studies in a range of other systems suggests that divergent temperature optima for different life-history/immune traits are not uncommon [32]. For example, research on butterflies and isopods demonstrated that overall baseline phenoloxidase activity was higher at cooler temperatures (butterflies: 10°C or 17.7°C; isopod: 19°C) than warmer temperatures (butterflies: 27°C or 34°C; isopod: 26°C) [27,28]. Further, Suwanchaichinda & Paskewitz [73] showed that *An. gambiae* melanization of Sephadex beads was highest at 24°C relative to 27°C and 30°C. The production of melanin is essential for many other physiological processes in addition to innate immunity, such as egg hardening and cuticular tanning [34], which may be an explanation for why the rate of humoral melanization is faster at lower temperatures.

Similarly, in immune responses of the mosquito *Ae. aegypti* against *E. coli*, both the defensin peptide and phenoloxidase colocalize at the sites of melanin deposition. In addition, they are often present in the same melanotic capsules [74], potentially explaining why *DEF1* expression follows the pattern of melanization. Linder et al. [29] demonstrated that overall expression of a diversity of immune genes (*Pgrp-LC*, *Cactus*, *Spatzle*) in *D. melanogaster* were upregulated in response to heat-killed bacterial challenge at 17°C relative to flies housed at 25°C and/or 29°C. Additionally, expression of heat-shock protein *Hsp83* was upregulated at both 17°C and 29°C relative to flies housed at 25°C, suggesting that heat-shock proteins may boost enzymatic efficiency at cooler temperatures in addition to high temperatures [29,75].

Phagocytic index and capacity were also higher in mosquitoes maintained at 18–24°C relative to warmer temperatures. So far as we are aware, there has been very little research examining temperature influences on phagocytosis in general. In monarch butterflies, the number of circulating haemocytes was greater at 10°C compared with warmer temperatures (27°C and 34°C). In ectothermic vertebrates, non-specific defences might play an important role in offsetting immune suppression at low environmental temperatures, while the specific immune system adapts. The rate of phagocytosis significantly increased with low environmental temperatures in tench (*Tinca tinca*) [76], channel catfish (*Ictalurus punctatus*) [77] and rainbow trout (*Oncorhynchus mykiss*) [78].

As with numerous other transcriptional studies, we have not linked temperature-induced variation in gene expression with functional resistance or vector competence, and it is possible that temperature might significantly modify post-transcriptional regulation. Thus, the effects of temperature on antimicrobial peptide production, nitric oxide enzyme activity and pathogen clearance should be investigated. Equally, we do not know how much melanin is required for pathogen killing, and hence whether the functional temperature optimum for melanization is at 18°C or 28°C (i.e. where we found the highest proportion of beads showing any level of melanization). Nonetheless, the interactions among temperature, the type of immune challenge and the time point at which mosquitoes are evaluated post-immune challenge clearly complicate interpretation of the many studies conducted under one set of conditions.

For instance, it is commonplace to infer importance of different elements of immune function by measuring fold differences in expression relative to some control baseline (e.g. [2,3,11]). In our study, it is clear that fold differences would differ substantially depending on the individual immune measure, nature of the controls, temperature and time point, yet the vast majority of expression/transcriptional studies ignore such complexities. Similarly, it is generally accepted that the immune gene families and pathways, and the associated mosquito immune responses implicated in resistance to the rodent malaria parasite (*P. berghei*) are different from those involved in defence against the human malaria parasite (*P. falciparum*) [1,58,79]. However, experiments on *P. berghei* are typically run at 19–21°C, whereas experiments on *P. falciparum* are run at around 27°C. Given the differential effects of temperature on immune responses, such as melanization and nitric oxide synthase across this range, it is unclear whether the reported differences in mosquito responses are actually parasite-derived, environment-derived or some combination of both. Further, it is unclear how temperature mediates interacting immune responses that experience diverse temperature optima, such as the potential reactivity of nitric oxide with components of the melanization response and phagocytosis [33]. It is quite possible that the relative importance of different immune mechanisms for controlling the same pathogen species varies with temperature. More broadly, with aspects of mosquito resistance being important for the success of insecticides [80,81], fungal biopesticides [82,83], biological larvicides [84] and prospective transgenesis, and paratransgenesis and transinfection tools in the field [85,86], the implications of complex temperature–immune interactions could be far-reaching. Our results highlight the need to begin framing vector immunity in the context of the ecologically variable world in which mosquitoes and parasites/pathogens interact.
